# The Contribution of the Limbus and Collagen Fibrils to Corneal Biomechanical Properties: Estimation of the Low-Strain In Vivo Elastic Modulus and Tissue Strain

**DOI:** 10.3390/biomimetics9120758

**Published:** 2024-12-13

**Authors:** Frederick H. Silver, Tanmay Deshmukh, Dominick Benedetto, Mickael Asfaw, Olivia Doyle, Nicholas Kozachuk, Kamryn Li

**Affiliations:** 1 Department of Pathology and Laboratory Medicine, RWJMS, Rutgers, The State University of New Jersey, Piscataway, NJ 08854, USA; 2 OptoVibronex, LLC, Ben Franklin TechVentures, Bethlehem, PA 18015, USA; tmd24895@gmail.com; 3 Center for Advanced Eye Care, Vero Beach, FL 32960, USAmha224@lehigh.edu (M.A.); ngk324@lehigh.com (N.K.); kml224@lehigh.com (K.L.)

**Keywords:** collagen fibrils, elastic modulus, shear stress, limbus, cornea, keratoconus, Bowman’s layer, biomechanics, keratoconus, cone formation

## Abstract

We have compared the biomechanical properties of human and porcine corneas using vibrational optical coherence tomography (VOCT). The elastic modulus of the cornea has been previously reported in the literature to vary from about several kPa to more than several GPa based on the results of different techniques. In addition, the formation of corneal cones near the central cornea in keratoconus has been observed in the clinic. Measurements of the resonant frequency and morphology of human and porcine corneas were used to evaluate the role of the limbus in corneal stabilization, the effect of Bowman’s layer, and the effect of collagen content on the low-strain corneal biomechanics. The results of these studies indicate that limbus stability plays an important anatomic role in preventing folding, corneal slippage, and cone formation. Machine learning studies of both human and porcine corneas indicate that Bowman’s membrane, like that of the collagen fibrils found in the anterior corneal stroma, contributes to the 110–120 Hz resonant frequency peak. Finite element and SOLIDWORKS models of normal and keratoconus corneas suggest that the deformation of the cornea is the highest at the central zone and is higher in keratoconus corneas compared to normal controls. VOCT results suggest that although collagen fibril slippage occurs first at the limbus, cone formation in keratoconus occurs centrally/paracentrally, where stress concentration and deformation due to intraocular forces are the highest. Cone formation occurs at the points of maximum curvature. Results of these studies indicate the elastic modulus of cornea fibrillar collagen dictates the corneal elastic modulus at low strains. These results suggest that tension in the cornea at the limbus results in deformation into the low modulus region of the J-shaped stress–strain curve, resulting in an in vivo strain of less than about 10%. We propose that tension in the cornea provides a baseline force that regulates corneal epithelial regeneration as well as corneal lamellae composition and matrix turnover.

## 1. Introduction

The cellular components of the cornea include epithelial cells, keratocytes, and endothelial cells [1,2,3]. Extracellular matrix components of the cornea include collagen fibrils and glycosaminoglycans in the stroma and circumferentially oriented collagen fibrils at the cornea–limbus interface of the human eye [4,5]. Circumferential anterior corneal stromal rigidity appears to be particularly important in maintaining corneal curvature and mechanical stability [6]. The cornea–limbus–scleral mechanical unit provides rigidity to the cornea and prevents cone formation [7]. The orthogonal layers found in the lamellae have been proposed to connect to collagen fibrils that make up the circumferential diamond-shaped pattern at the interface between the collagen fibrils of the limbus and stroma [4,5,7]. In the absence of mechanical connections at the interface between the cornea and sclera, corneal delamination and cone formation would occur during blunt trauma and other ocular diseases [7,8]. Recent indirect evidence suggests that slippage seen in the mechanical connections and changes in the elastic modulus at the cornea–limbus interface promotes central corneal cone formation in keratoconus patients, leading to visual changes [8]. Therefore, the determination of the biomechanical properties of corneal components is an important criterion for maintaining normal vision, early diagnosis of degenerative corneal diseases such as keratoconus, and longitudinal follow-up and treatment [7,8].

A variety of methods have been used to measure the mechanical properties of the cornea. Measurement of corneal biomechanical properties in vivo has been conducted using the Ocular Response Analyzer (ORA; Reichart Ophthalmic Instruments, Buffalo, NY, USA) [9,10,11], Corvis ST (Oculus, Wetzlar, Germany) [10], Brillouin optical microscopy [12,13], magnetic resonance elastography [14], optical coherence (OCE) elastography [15,16,17], and vibrational OCT (VOCT) [7,8]. Alterations in biomechanical properties of the cornea occur in degenerative diseases, such as keratoconus, connective tissue diseases, including Marfan disease, and iatrogenic corneal ectasia [10,16]. They also have been reported to be associated with the onset of myopia and glaucoma [18]. Measurements made using these methods have led to the conclusion that changes in the biomechanical properties of the cornea occur in corneal diseases; however, the modulus (stiffness) values predicted from the results of these studies vary by several orders of magnitude [7]. This variation in measured modulus values of cornea using different methods makes assessing the pathogenesis of corneal diseases difficult in relationship to the changes in the tissue components such as collagen [8]. Therefore, there is a need to develop methods to measure corneal modulus values that are consistent with the moduli of other collagenous soft tissues, such as skin, that are primarily composed of collagen fibrils and fibers.

We have developed a technique termed vibrational optical coherence tomography (VOCT) that uses infrared light and sound to apply a mechanical transverse deformation to the cornea to measure the resonant frequency and elastic moduli of the cornea and other ocular components [7,8,19,20,21,22]. While both porcine and human eyes have similar resonant frequencies for the corneal components, they differ in the resonant frequency peak heights near the frequencies of 80, 110–120, 140–150, and 240–250 Hz [7,8,19,20,21,22]. The 60–80 Hz peak is reported to be associated with corneal epithelial cells and keratocytes [7,8,19,20,21,22]; the 110–120 Hz and 140–150 Hz peaks are associated with the collagen lamellae [18,19,20], and the 240–250 peaks are associated with the scleral–limbus–corneal junction [7,8,19,20,21,22]. Results of studies on whole porcine corneas and measurements on dissected anterior segments of the cornea indicated that the 110–120 Hz and 140–150 Hz resonant frequency peaks reside in the anterior segment of the cornea since the peak heights decreased after the anterior parts of the cornea were removed [19,20,21,22]. A recent VOCT study concluded that the cornea and limbus mechanical properties were altered in patients with keratoconus (KC), with the modulus (stiffness) of the central cornea decreasing in KC while that in the lower cornea increasing when compared to control corneas [8].

The purpose of this paper is to report the results of VOCT studies on porcine and human corneas to study the role of the limbus in contributing to corneal tensile behavior, to determine the low-strain elastic modulus of Bowman’s layer, and to explain the reasons for central/paracentral cone formation in KC when collagen fibril slippage appears to occur at the cornea–limbus interface.

## 2. Materials and Methods

### 2.1. Weighted Displacement Versus Frequency Plots

The displacement measurements as a function of acoustic sound frequency were made using a Lumedica Labscope 2.0 modified to make displacement measurements on raw images. The transverse sample displacement was determined in phase with the acoustic sound using special software developed as discussed previously [19,20,21,22]. Mechanovibrational VOCT spectra on human corneas were collected on 41 normal control eyes and 22 eyes from subjects diagnosed with stages I through IV KC, as previously reported [8]. IRB approval for human studies was obtained from Wills Eye Hospital, Philadelphia, PA. Patient informed consent was collected on controls and KC subjects [8]. The VOCT data were collected by measuring the displacement versus frequency of sinusoidal acoustic sound applied to the cornea, as described previously, between 50 and 250 Hz [7,8,19,20,21,22]. All displacements were normalized by dividing by the speaker displacement measured in the absence of the sample. Measurements from the eyes of patients exhibiting the following stages of KC were studied: Stage I (N = 2); stage II (N = 9); stage III (3); and stage IV (8). None of the patients underwent collagen crosslinking prior to making the VOCT measurements. Measurements were made by focusing the sound and infrared light beam centrally and then on the inferior cornea above the limbus (at 6 o’clock), with the subject looking upward.

Measurements were also made on 12 intact porcine eyes and 9 porcine corneas after dissection with and without an attached thin scleral ring. Some porcine corneas were frozen at −20 °C for one week before testing. Freezing did not affect the peak locations in the weighted displacement versus frequency plots.

### 2.2. Collection of OCT Images

OCT image collection on human and porcine corneas was conducted using a Lumedica Spectral Domain OQ 2.0 Labscope (Lumedica Inc., Durham, NC, USA) operating in the scanning mode at a wavelength of 840 nm. The device generates a 512 × 512-pixel image with a transverse resolution of 18 μm and an A-scan rate of 13,000/s. The images were used to locate where the VOCT measurements were made on each cornea. Special software was used to analyze single OCT raw images at a fixed position for VOCT measurements. OCT images were color-coded to improve visualization of the epithelium and collagen fibrillar components, as described previously [7,8].

The resonant frequency of each tissue component was defined as the frequency at which the maximum in-phase displacement was observed in the amplitude data. The amplitude of the weighted displacement versus frequency plot was used to define the resonant frequencies of the tissue components. The measured resonant frequencies were converted into elastic modulus values using a calibration equation (Equation (1)) developed based on in vitro uniaxial mechanical tensile testing and VOCT measurements on soft tissues made at the same time, as reported previously [7,8,19,20,21,22]. The resonant frequency of each sample was determined by measuring the displacement of the tissue resulting from applied sinusoidal audible sound driving frequencies ranging from 50 Hz to 250 Hz (humans) and from 50 to 300 (porcine corneas) in steps of 10 Hz [7,8,17,18,19,20]. The location of the peaks on some of the samples differed by as much as 10 Hz since the measurements were made at intervals of 10 Hz. The peak frequency (the resonant frequency), fn, was defined as the frequency at which the elastic displacement was maximized after the vibrations due to the speaker being removed.

E × d = 0.0651 × fn^2^ + 233.1 (1)

**Figure 1:**
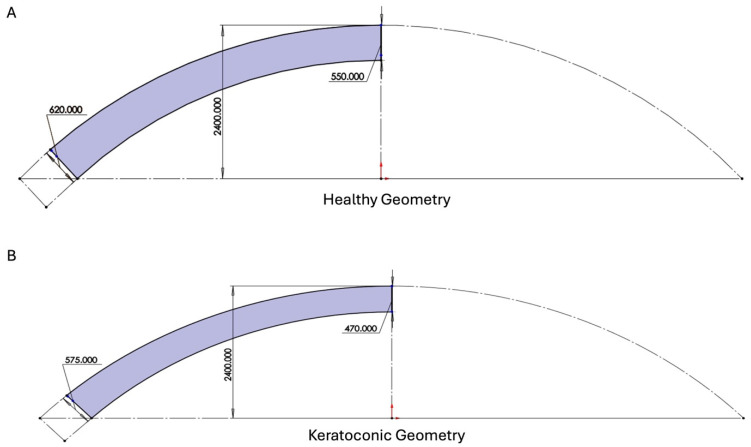
SOLIDWORKS model with the dimensions used in the analysis of healthy (**A**) and keratoconic corneas (**B**). All numbers shown are in micrometers. Typical dimensions used in the model were obtained from a previous publication [8].

**Figure 2:**
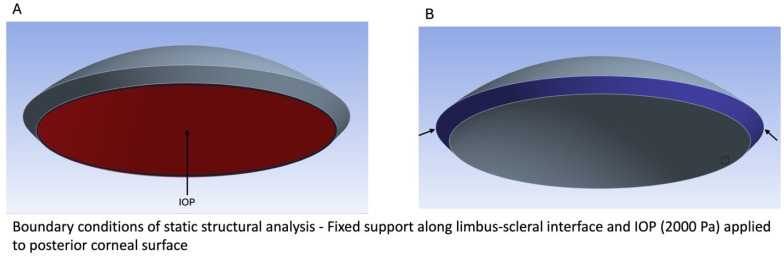
Diagram illustrating the dimensions and boundary conditions of IOP (**A**) and fixed support (**B**) used in the finite element analysis.

Since soft tissues have a density very close to 1.0, Equation (1) is valid for most soft tissues found in the body where the thickness d is in m and is determined from OCT images; fn^2^ is the square of the resonant frequency, and E is the tensile elastic modulus (stiffness) in MPa as discussed previously [7,8,19,20,21,22]. Equation (1) is an empirical equation used to calculate the elastic modulus based on calibration studies. The stiffness of soft collagenous tissues like the skin is unchanged in the low modulus region of the J-shaped stress–strain curve, which occurs at strains up to about 10% [23]. Models used to evaluate corneal behavior are shown in Figure 1 and Figure 2.

**Figure 3:**
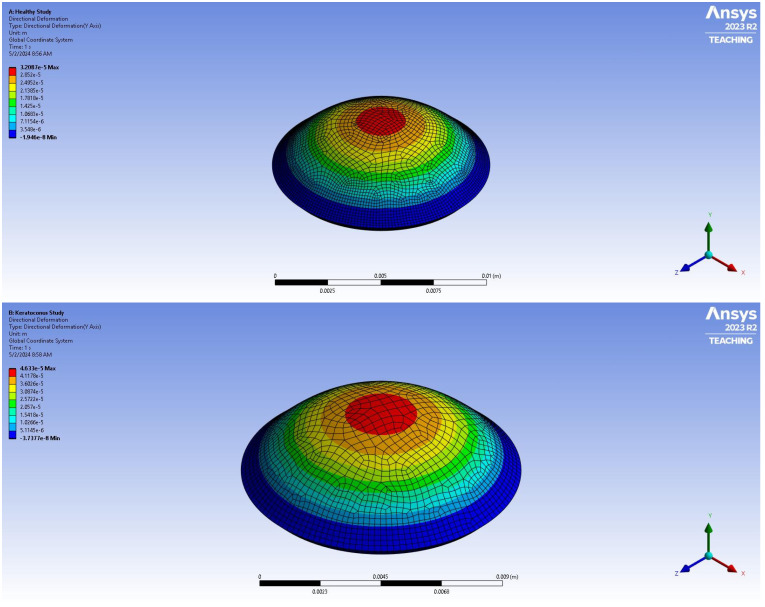
A map of estimated corneal displacement versus location color-coded to reflect the location of maximum displacement in red at the center for normal cornea (**A**) and for a keratoconus cornea (KC) (**B**) cornea. The plots were constructed from average modulus data for patients with KC stages I to IV [8]. The maximum displacement in each model occurs at the central point. Deformation in the central KC cornea averaged 1.443 times that of the normal corneas for all stages of KC. Note that the force of gravity or other forces influencing the deformation of the cornea were not considered in the modeling. Cone formation in KC corneas occurs centrally/paracentrally, while collagen fibril slippage occurs at the limbus–corneal junction. The model was constructed using 1024 elements, and uniform deformation of all elements was assumed to be equal. The mechanical properties of all elements were held constant. The central edge of the cornea was assumed to be unconstrained, while the outer edges were constrained from movement by connection to the stiffer limbus that surrounded the cornea. The relationship between the elastic modulus and the strain was assumed to be linear at low strains. The model assumes linear behavior of an isotropic material with a Posson’s ratio of 0.42 as indicated in Table 1 [29].

The average modulus was estimated by calculating the product of the weighted displacement peak height (see Figure 3) and the component modulus values and then dividing by the sum of the peak heights. The resonant frequencies of the individual peaks have been identified previously [19,20,21,22]. The mean cornea modulus is, therefore, an average weighted by the amount of light reflected from the individual tissue components back to the detector. The average corneal modulus used in this study included measurements for all stages of KC, as reported previously [8].

### 2.3. Porcine Eye Dissection Experiments

Whole porcine eyes were obtained from Spear Products Inc. (Coopersburg, PA, USA). Eyes were tested either within 2.0 h of harvesting or after freezing for at least 7 days at −20 °C and then thawing at RT. During the dissection of porcine eyes, the fat and other extraneous tissues were removed. Once the extra tissue surrounding the eyes was removed, the globes were left intact, or the cornea was either cut with a razor blade through the scleral–limbus junction, leaving a small ring of the sclera, or cut through the cornea, removing the attached sclera–limbus junction. Color-coded OCT images, weighted displacement versus frequency measurements, and elastic moduli were made on the specimen with and without an attached scleral ring, as described previously [19,20,21,22].

### 2.4. Machine Learning Model for Bowman’s Layer Classification

A machine learning model was developed to ascertain the resonance frequency of Bowman’s layer. Since human corneas contain Bowman’s layer and porcine corneas do not, a comparison of the location of the resonant frequency peaks of human and porcine corneas was used to identify the resonant frequency of Bowman’s layer. The dataset comprised 40 sets of VOCT weighted displacement versus frequency data for human and porcine eyes, encompassing VOCT readings from 50 Hz to 250 Hz in 10 Hz increments. Two distinct machine learning algorithms, Variational Bayesian Gaussian Mixture Model and Support Vector Clustering, were employed to ensure robust and accurate results [24,25]. Before inputting the data into the machine learning model, the data were processed. The initial step involved normalizing the data by dividing it by the vibrations due to the speaker without the sample present. The weighted displacement versus frequency plot for each cornea was normalized by dividing by the largest peak in each data set. This converted the data into a range between 0 and 1. Normalization was used to compare data collected at each frequency on different samples.

The first machine learning algorithm applied was the Variational Bayesian Gaussian Mixture Model (VBGMM), an unsupervised clustering algorithm combining Gaussian Mixture Models with Bayesian methods and variational inference [24]. The input for this algorithm was all data points for a single frequency, run for each frequency, in which the VBGMM processed the data points with a parameter specifying the creation of two clusters. The output yielded classification labels (0 or 1) for each data point, representing human or porcine clusters. The model’s accuracy was determined by the ratio of correct classifications to the total number of total classifications. The results provided accuracy percentages for each frequency, with the highest accuracy indicating the resonance frequency of the Bowman’s layer, a key feature to distinguish between humans and porcine corneas.

To validate the findings derived from the Variational Bayesian Gaussian Mixture Model (VBGMM), a secondary machine learning algorithm, namely, Support Vector Clustering (SVC), was used [24]. Functioning as a supervised learning clustering algorithm, SVC aims to identify a hyperplane that maximizes the margin between cluster points. The leave-one-out approach was used for the SVC. In this approach, the input was all the data points for each frequency, with one frequency being left out. For example, for the analysis at 50 Hz, only the 60 Hz–250 Hz data were used as the frequency inputs. Like VBGMM, 2 clusters were also specified to be found by the algorithm. This process was iterated through all frequencies, where the data points were clustered using each frequency. Since SVC is a supervised learning algorithm, the data were split into 80% training data, and 20% testing data and five-fold cross-validation were used. Using 5-fold cross-validation, each fold was tested to remove any bias. Additionally, this was run repeatedly to ensure the best accuracy by having different combinations for each fold for different runs. The SVC produces clustering results for each data point, in which the total accuracy is calculated by dividing the number of correctly predicted points in a cluster by the total number of points. The results presented accuracy percentages for each frequency when excluded, indicating the resonance frequency of Bowman’s layer as the one with the lowest accuracy. The decrease in overall accuracy in the absence of one frequency data signified that the identified output frequency contributed to distinguishing between human and porcine clusters for Bowman’s layer.

### 2.5. Finite Element Analysis

A finite element analysis (FEA) with 1024 elements was used to simulate how a model of the cornea reacted to various applied forces and boundary conditions [26]. Static structural analysis was used to simulate the deformational differences between KC and healthy corneas. In static structural analysis, static boundary conditions were used to analyze how the cornea responded to an applied load. To study the deformational differences between healthy and KC corneas, the maximum axial deformation was calculated under the force of intraocular pressure at different points on the cornea. The material properties of the cornea reported from previous VOCT studies were used, as well as corneal thicknesses for normal and KC eyes [8]. The material properties used in the FEA, including density, elastic modulus, and Poisson’s ratio, are listed in Table 1.

SOLIDWORKS was used to create the 3D models of both the KC and healthy corneas. In KC, the peripheral corneal thickness increases, and the central corneal thickness decreases. The precise dimensions of both the keratoconic and healthy corneal 3D models described were obtained from Pentacam and OCT measurements [8]. The dimensions of the normal and peripheral corneas and those of KC corneas are shown in Figure 1.

The boundary conditions of the static structural model were made such that they reflected the conditions of an in vivo cornea. The peripheral face of the cornea was selected to be a fixed support, as this section of the cornea would be attached to the limbus. Additionally, an internal pressure of 2000 Pascals (20 mm Hg) was applied to the posterior surface of the cornea, which represented the normal intraocular pressure (IOP), as diagrammed in Figure 2.

## 3. Results

Figure 1 and Figure 2 show the model used for the finite element analysis created using SOLIDWORKS to illustrate the corneal deformation as a function of the position of the cornea. As illustrated in Figure 1, the cornea is thicker at the edges as opposed to the center, with the normal cornea being thicker than the corneas from the KC patients. This results in the deformation of the cornea being higher at the center in the KC cornea versus the control cornea (Figure 3). The deformation is also higher at the center compared to the edge of both normal and KC corneas. Figure 3 shows the FEA model of corneal displacement as a function of location on the corneal surface.

**Figure 4:**
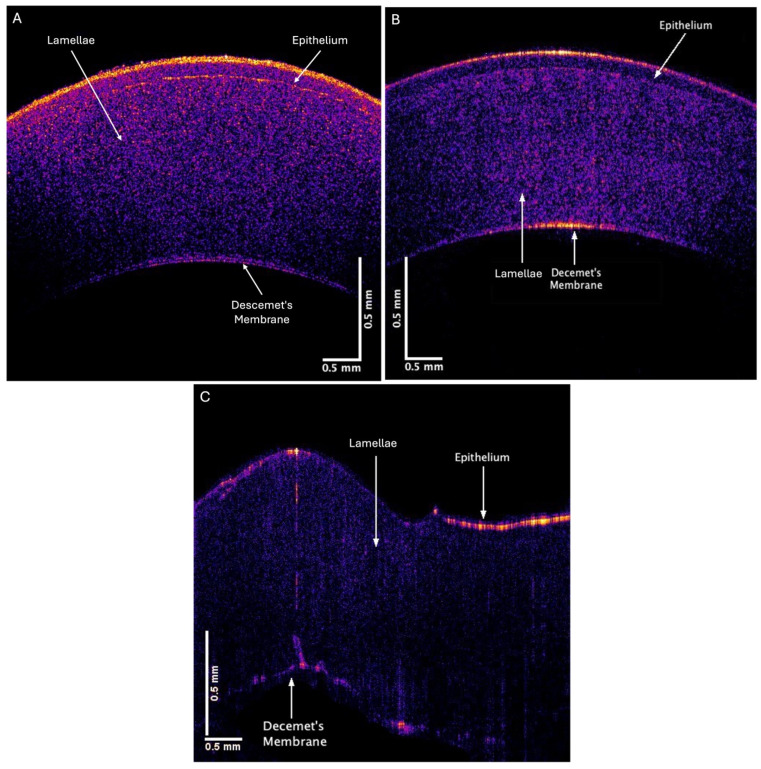
Typical OCT color-coded images of porcine corneas from (**A**) whole eyes after removal of extraneous tissue surrounding the globe, (**B**) porcine cornea with a sclera ring, and (**C**) porcine cornea without a scleral ring. The presence of a scleral ring containing limbus prevents folding of the cornea.

**Figure 5:**
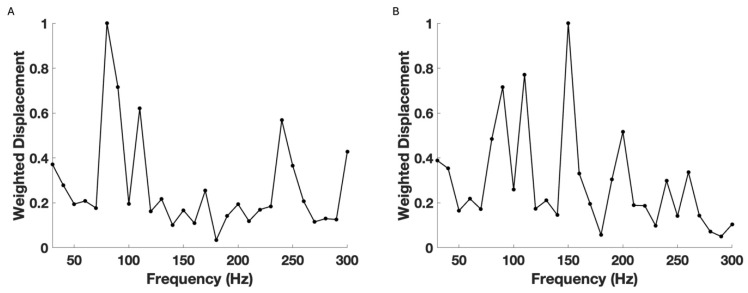

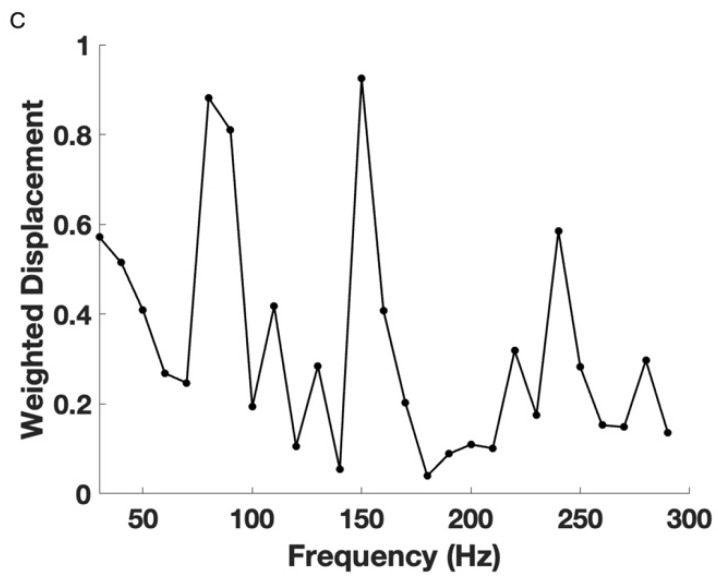
Typical plots comparing the normalized weighted displacement versus frequency plots for porcine cornea when the measurements were made on (**A**) the whole porcine globe focused on cornea, (**B**) excised porcine cornea with a scleral ring, and (**C**) excised cornea without a scleral ring (**C**). Note that the locations of the peaks in B and C are similar, with some differences in the peak heights. The peak heights in A are influenced by vibrations from the sclera in addition to vibrations from the cornea and limbus. The peaks at 80, 110–120, 140–150, and 240–250 Hz have been assigned to epithelial cells and keratocytes (80 Hz), anterior and posterior collagen lamellae (110–120, 140–150 Hz), and limbus and sclera (240–250 Hz) [7,8].

Figure 4 shows color-coded OCT images of porcine corneas from an intact globe (A), an excised pig cornea with a thin ring of sclera (B), and an excised pig cornea that has had the sclera–limbus junction removed during dissection (C). Porcine eyes are similar to human eyes, with the main difference being that the porcine cornea does not have Bowman’s layer [30]. The removal of the sclera–limbus junction from the cornea leads to the formation of folds in the cornea and retraction from the edges. Figure 5 shows plots of weighted displacement versus frequency for porcine corneas from a whole pig globe (A), a cornea with an intact scleral–limbus junction (B), and a cornea lacking the scleral–limbus junction (C). Although the peak heights are different for whole eyes and isolated corneas, the location of the peaks is similar in corneas with and without the scleral–limbus junction.

**Figure 6:**
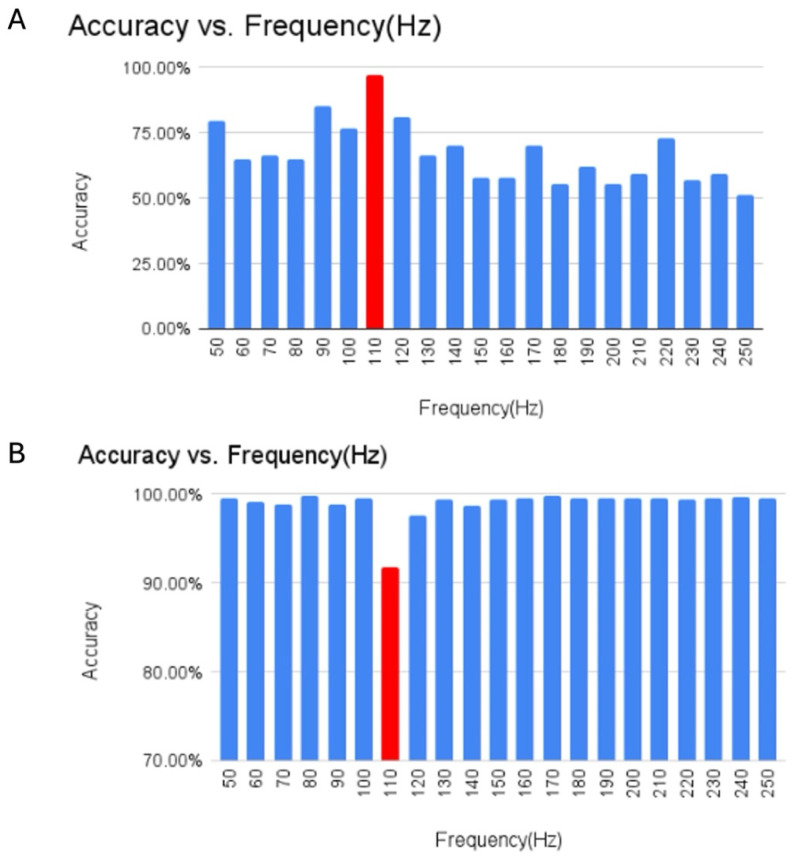
Machine learning results using (**A**) VBGMM and (**B**) SVC models show the 110 Hz peak to be the distinguishing factor between human and porcine eyes. Porcine corneas do not contain Bowman’s layer, while human eyes have one. Bowman’s layer appears to contribute to the 110–120 Hz peak identified in the anterior portion of the cornea. Note that both models agree that the Bowman’s layer contributes to the 110 Hz peak, which is equivalent to a modulus of about 2.5 ± 0.25 MPa based on a measurement of the resonant frequency to an accuracy of ±10 Hz [7,8].

As seen in Figure 6, the machine learning models developed to distinguish between human and porcine corneas indicated that the 110 Hz resonance frequency peak was diminished in porcine eyes compared to human eyes. Figure 6A shows the result of the VBGMM, illustrating that the 110 Hz peak resulted in the highest accuracy of classifying human and porcine data. The average accuracy varied from 0.9216 ± 0.0963 at 110 Hz to 0.9940 ± 0296 at 210 Hz. This is the distinguishing factor between humans and pigs found in this model. Figure 6B illustrates that when the 110 Hz peak is left out, the accuracy of classifying human and porcine data decreases based on the SVC model. Based on previous reports and these results, Bowman’s layer has a low-strain elastic modulus of 2.5 +/− 0.25 MPa [7,8,22]. These results support the conclusion that the Bowman’s layer is a part of the 110–120 Hz peak [7,8].

Results for the finite element model using SOLIDWORKS gave the following results. The maximum deflection in normal and keratoconus corneas occurred at the center of the cornea (Figure 3). The deflection in the central KC cornea was 1.443 times that of the normal cornea, suggesting that cone formation was initiated at or near the central region where the maximum deformation and stress concentration occurred and the corneal curvature changed rapidly. Cone formation does not form at the periphery of the corneal, where the collagen slippage appears to initiate.

## 4. Discussion

Measurement of the corneal elastic modulus has been a subject of great interest for many years, and changes have been associated with the development of ocular diseases [18,19]. Determination of changes in the corneal elastic modulus may assist in the early diagnosis of several corneal diseases, including KC, corneal ectasia, and diseases not traditionally associated with the cornea, such as myopia and glaucoma [31,32]. Several studies have reported corneal modulus values varying from as little as about 72 kPa [32] to about 2.7 GPa by Brillouin light scattering [33]. This large range of values reported is due to the use of different testing procedures and differences in how the viscoelasticity of the cornea was treated in the data analysis. It is, therefore, important to determine the exact value of the elastic modulus of the cornea in vivo and how it compares to the modulus of other soft tissues containing similar collagen contents. Knowing the baseline value of the normal elastic modulus is essential to evaluate changes that occur in different corneal diseases.

While the elastic modulus of isolated cells has been reported to be from 100 Pa to 100 kPa [34], epithelium offers little resistance to collagen fibrillar slippage and, therefore, cone formation in the cornea. Individual cells are reported to be stiffer than cells in a confluent monolayer [35], suggesting that intercellular forces do not appear to be involved in direct limitation of corneal cone formation. In KC shear stresses of a few Pa, significantly influence the cell state [36]. Corneal epithelial cells exposed to low amounts of shear stress demonstrated more prominent filamentous elongated actin filaments and higher rates of migration and proliferation, following 24 h of low-shear stress exposure [37]. While the corneal epithelium provides some contribution to the mechanical properties of the cornea, it is the collagen fibrils that provide most of the support to counter tensile stresses that prevent the cornea from folding when the limbus is detached. It is, therefore, the interactions between the components of the extracellular matrix that withstand the effects of gravity, intraocular pressure, and accommodation. The connections within the cornea–limbus–scleral mechanical unit, including the circumferential collagen fibrils found at the cornea–limbus junction, appear to stabilize corneal curvature and shape and provide mechanical connections with the corneal lamellae, preventing collagen fibril slippage and cone formation.

Machine learning results reported in this paper suggest that Bowman’s layer contributes to the 110–120 resonant frequency peak previously reported, which has an elastic modulus of about 2.5 ± 0.25 MPa based on measurement of the resonant frequency to an accuracy of ±10 Hz [7,8]. Bowman’s layer is found beneath the epithelial basement membrane and is primarily composed of type I collagen fibrils with few cells in the adult [38]. The results of one study showed that Bowman’s layer did not contribute significantly to mechanical stability within the cornea [39]. It is concluded in this study that Bowman’s layer has a similar stiffness to the other collagen fibrils in the anterior portion of the cornea and does not enhance the mechanical stability of the cornea.

The collagen fibrils in the limbus have been reported to be stiffer than the collagen fibrils found in lamellae of both porcine and human eyes [19,20,21,22]. This observation suggests that the tension in the cornea is generated by the rigidity of the cornea–limbus junction, as reported previously [22]. This rigidity can be explained by the mechanical connections between the circumferential collagen fibers at the cornea periphery and their connections to the collagen lamellae found centrally, as described previously [5,6,7,8,22]. This is important since any shape or surface change to the cornea associated with diseases would affect visual acuity. However, even though collagen slippage appears to occur first at the limbus–collagen interface, cone formation in KC occurs centrally/paracentrally. Our modeling results suggest that cone formation is a result of increased deformation where the corneal curvature is maximized. This observation is consistent with modeling performed by Shih et al. [40], who reported that the stress concentration was maximized at the central cornea, and, therefore, the strain would also be maximized at this point. This observation is consistent with the results reported in this paper.

Decreases in the central cornea stiffness in KC lead to increased deformation of the central cornea and are accompanied by slippage of the collagen fibrils at the scleral–limbus junction. This slippage and central corneal deformation lead to cone formation centrally. In comparison, VOCT measurements on the lower cornea of KC patients suggest that it exhibits an increased stiffness compared to control subjects [8]. Stiffening of the lower cornea in KC patients may be a protective compensation mechanism to decrease the rate of cone formation throughout the cornea. Without this increase in the lower corneal modulus, mechanical failure would be enhanced in KC.

Limitations to the finite element model include the assumption that the cornea is a homogeneous and isotropic material that exhibits a linear stress–strain behavior in the low-strain region. It also assumes that the intraocular pressure uniformly affects the internal corneal surface and that the stress at the limbus corneal junction is uniformly applied around the outside of the cornea.

The modulus of the human cornea varies greatly depending on the technique used and the assumptions made, as reviewed previously [41]. We have reported that the average modulus of normal human and porcine corneas is about 2 MPa (lower cornea) to 3 MPa (central cornea) [8]. This is like the elastic modulus of purified collagen and dermal collagen in human skin. That value is reported to be 2.50 MPa based on VOCT results measured in vivo [7,8,22]. This value is like the low-strain modulus of isolated human dermis at strains of up to 10% measured in vitro [42]. This modulus value is much lower than the high strain value of the elastic modulus of the dermis of 16.46 MPa found for strains above 20% [42]. These results suggest that the low-strain modulus of the human cornea in vivo is like that of collagen in the human dermis, and the in vivo strain appears to be less than 10%. This indicates that the cornea, like skin, operates in vivo in the low-strain region of the J-shaped stress–strain curve. The human corneal collagen content is about 71% by dry weight [43], similar to that of human skin (75%) [44]. Therefore, even though the collagen fibril sizes are different in the cornea and skin, the low-strain elastic moduli are similar; both skin and cornea operate in the low modulus region of the J-shaped stress–strain curve in vivo. Tension and energy that are a result of stretching both skin and cornea in vivo may be involved in maintaining tissue shape and providing a baseline for regulating the level of mechanotransduction in the resting state of these tissues [45]. Any perturbations in the tension and energy stored in the cornea due to modulus changes may either down- or up-regulate mechanotransduction, leading to different ocular diseases. While the strength of different tissues containing collagen fibrils and fibers may differ based on differences in collagen fibril diameters, the low-strain modulus appears to be similar and based on the collagen content. Thus, maintenance of a uniform collagen stiffness in tissues may provide a baseline for mechanotranduction that supports normal tissue metabolism and limits differences at interfaces between soft tissues. Differences in tissue moduli at interfaces lead to failure at graft–host interfaces in hernia replacement surgery and result in intimal hyperplasia in vascular grafts.

Other limitations of this study include basing the forces used in modeling solely on intraocular pressure and not including gravity and extraocular muscle forces that exist in the eye. This model also uses a linear relationship for stress and strain (deformation), which assumes that the deformation is limited to the low-modulus region of the stress–strain curve.

The results presented in this paper suggest that cone formation in KC corneas occurs near the center, where the cornea is the thinnest and has the maximum curvature, which is consistent with clinical observations reported by Roberts et al. [46] that corneal stress is greatest at the thinness region with the greatest curvature.

## 5. Conclusions

We have studied the biomechanical properties of human and porcine corneas both in vivo and in vitro using VOCT. The results of these studies indicate that the limbus–corneal junction is important in preventing corneal slippage and folding in vivo. Machine learning studies indicate that Bowman’s membrane contributes to the 110–120 Hz resonant frequency peak like the collagen fibrils found in the anterior corneal lamellae. Finite element models of normal and KC corneas suggest that the deformation of the cornea is highest at the central zone and that it is higher in KC corneas compared to normal controls. These results suggest that cone formation in KC corneas occurs centrally/paracentrally, where stress concentration is the highest at the points of maximum curvature, even though collagen fibril slippage appears to occur at the limbus–collagen junction. Finally, the elastic moduli of cornea and skin are similar and average about 2.5 MPa even though the collagen fibril diameters are not similar. Both skin and cornea operate in vivo in the low modulus region of the J-shaped stress–strain curve. The tension derived from forces exerted on the eye stretches the cornea into the low modulus region of the J-shaped stress–strain curve, resulting in an in vivo strain of less than 10%. The similar modulus values for cornea and skin in vivo appear to depend only on collagen content, while the strength of different collagenous tissues may be dependent on collagen fibril and fiber sizes.

## Figures and Tables

**Table 1:** Parameters used in the finite element model of corneal deformation with parameters previously reported [8,27,28,29]. IOP stands for intraocular pressure in the table.

Model	Elastic Modulus (MPa)	Poisson’s Ratio	Density (g/cm)	IOP (Pa)
Keratoconic	2.4	0.42	1.038	2000
Healthy	3.1	0.42	1.038	2000

## Data Availability

Data are available at optovibronex.com (accessed on 6 December 2024).
